# Kisspeptin as a test of hypothalamic dysfunction in pubertal and reproductive disorders

**DOI:** 10.1111/andr.13843

**Published:** 2025-01-20

**Authors:** Aureliane C. S. Pierret, Aaran H. Patel, Elisabeth Daniels, Alexander N. Comninos, Waljit S. Dhillo, Ali Abbara

**Affiliations:** ^1^ Section of Endocrinology and Investigative Medicine Imperial College London London UK; ^2^ Department of Endocrinology Imperial College Healthcare Trust London UK

**Keywords:** hypogonadism, kisspeptin, pubertal disorders, reproductive disorders

## Abstract

The hypothalamic–pituitary–gonadal axis is regulated by the gonadotropin‐releasing hormone pulse generator in the hypothalamus. This is comprised of neurons that secrete kisspeptin in a pulsatile manner to stimulate the release of GnRH, and, in turn, downstream gonadotropins from the pituitary gland, and subsequently sex steroids and gametogenesis from the gonads. Many reproductive disorders in both males and females are characterized by hypothalamic dysfunction, including functional disorders (such as age‐related hypogonadism, obesity‐related secondary hypogonadism, hyperprolactinemia, functional hypothalamic amenorrhea and polycystic ovary syndrome), structural pathologies (such as craniopharyngiomas or radiation or surgery‐related hypothalamic dysfunction), and pubertal disorders (constitutional delay of growth and puberty and congenital hypogonadotropic hypogonadism). However, in many of these conditions, the relative contribution of hypothalamic dysfunction to the observed hypogonadism is unclear; as to date, there is no direct method of evaluating hypothalamic reproductive function in humans. Indeed, it is not possible to directly measure gonadotropin‐releasing hormone levels in the hypothalamo‐pituitary portal vessels, such that secondary (i.e., pituitary dysfunction) and tertiary (i.e., hypothalamic dysfunction) hypogonadism are often conflated as one entity. In this review, we examine the evidence for the use of kisspeptin as a method of directly evaluating hypothalamic reproductive dysfunction, and deliberate its potential future role in the evaluation of pubertal and reproductive disorders.

## 1 INTRODUCTION

Reproductive function is controlled by the hypothalamic–pituitary–gonadal (HPG) axis in both males and females. The hypothalamus secretes gonadotropin‐releasing hormone (GnRH) in a pulsatile manner, which, in turn, stimulates the release of gonadotropins, that is, luteinizing hormone (LH) and follicle‐stimulating hormone (FSH) from gonadotroph cells in the anterior pituitary gland.^1^ These gonadotropins act on the ovaries in women to regulate folliculogenesis, ovulation, and secretion of female sex steroids (estrogen and progesterone), and in the testes in men to regulate spermatogenesis and the secretion of male sex steroids (testosterone).^2^

Pulsatility of GnRH release is essential for stimulation of the reproductive axis, as evidenced by pulsatile but not continuous infusions of GnRH being able to restore gonadotropin secretion in rhesus monkeys with hypothalamic lesions.^3^ GnRH secretion is regulated by a neurohumoral network termed the “GnRH pulse generator,”^3^ primarily composed of a population of neurons co‐expressing “kisspeptin (KP), neurokinin B (NKB), and dynorphin” (KNDy) in the arcuate nucleus (ARC) of rodents (analogous to the infundibular nucleus of humans).^4^, ^5^ Kisspeptins are peptides encoded by the *KISS1* gene in humans; the likely native form is kisspeptin‐54 (KP54) comprised of 54 amino acids, which can be further cleaved to shorter kisspeptins of 14, 13, or 10 amino acids.^6^ These act on the kisspeptin receptor encoded by the *KISS1R* gene, located on GnRH neurons where they stimulate GnRH release.^6^, ^7^ KP54 has a longer half‐life than KP10 (32 vs. 4 min), and stimulates a more potent and sustained LH response than KP10 after bolus administration.^8^ KP54, unlike KP10, also appears to cross the blood–brain barrier and thus be able to act on GnRH neuronal cell bodies as well as nerve terminals at the median eminence.^8^ The importance of kisspeptin in stimulating the HPG axis and normal reproductive function is demonstrated by loss of function variants in *KISS1R* and *KISS1* genes resulting in congenital hypogonadotropic hypogonadism (CHH),^9^, ^10^, ^11^ whilst gain‐of‐function variants in *KISS1R* result in precocious puberty because of premature activation of the HPG axis.^12^

Hypothalamic dysfunction is central to many reproductive disorders in both men and women. Some of these disorders are functional, that is, do not have a clear structural/organic basis: for example, age‐related hypogonadism^13^; obesity‐related hypogonadism^14^, ^15^; hypothalamic hypogonadism (including functional hypothalamic amenorrhea [FHA] in women and the equivalent condition in men, relative energy deficiency syndrome [RED‐S] in athletes,^16^, ^17^ hyperprolactinemia, and drug‐induced causes such as hypogonadism induced by opiate use); and pubertal disorders such as constitutional delay of growth and puberty (CDGP).^18^ In addition, there are organic/structural causes of hypothalamic hypogonadism: such as post‐radiation or craniopharyngioma, and CHH.^18^, ^19^ Polycystic ovary syndrome (PCOS), a common cause of anovulation and oligomenorrhea causing reproductive dysfunction in women, is not characterized by hypogonadism as estradiol (E2) levels are typically preserved, especially in the context of oligomenorrhea; however, it is associated with abnormally increased hypothalamic function and anovulation.^20^

There is currently no direct method of evaluating hypothalamic reproductive function in humans, and thus of determining the contribution of hypothalamic dysfunction in reproductive disorders.^13^ This is due to the inability to directly measure GnRH levels from the hypothalamo‐pituitary portal vessels. As an indirect measure, the response to graded titration of a GnRH receptor antagonist has been used in a research context.^21^ Recently, kisspeptin has emerged as a direct specific stimulus of hypothalamic GnRH secretion. In this review, we examine the potential for kisspeptin as a method of directly evaluating hypothalamic function in reproductive and pubertal disorders associated with perturbed hypothalamic function.

## 2 CONGENITAL HYPOGONADOTROPIC HYPOGONADISM AND DELAYED PUBERTY

### 2.1 Congenital hypogonadotropic hypogonadism

CHH is a rare condition characterized by a failure of development of pulsatile GnRH secretion resulting in a lack of initiation of puberty with subsequent infertility.^19^ When CHH is combined with anosmia (as it is in approximately half of cases), it is termed Kallmann’ syndrome, which results from impaired embryonic migration of GnRH‐secreting neurons, or impaired GnRH secretion.^19^, ^22^ Variants in over 30 genes have been identified as causes of CHH, although up to half of cases do not have an identified genetic basis identified to date.^19^ Affected genes are involved in GnRH neuron fate specification, migration, neuroendocrine secretion and homeostasis; or cause gonadotroph defects.^19^ Up to one fifth of patients can have abnormalities in multiple genes called oligogenicity. Loss‐of‐function variants in *KISS1R* and *KISS1* genes have been found to be causes of CHH.^9^, ^10^, ^11^ Indeed, the reproductive impact of kisspeptin was first discovered in a family with CHH harboring an inactivating variant in *KISS1R*.^10^ Notably, most patients with CHH have a markedly reduced/absent LH and FSH response to stimulation with an IV bolus of kisspeptin, and thus kisspeptin could have clinical utility to identify patients with CHH.^23^

By contrast, many patients with CHH can still respond to stimulation with GnRH, and thus the gonadotropin response to kisspeptin is more specific and accurate at identifying CHH than GnRH. Indeed, the LH response to KP54 was shown to fully discriminate between adult men with CHH (*n* = 21) and healthy eugonadal men (*n* = 21) with an area under the receiver operating characteristic curve (AUROC) of 1.0 (95% CI: 1.0–1.0), whereas the response to GnRH was less discriminatory (AUROC = 0.88, 95% CI: 0.76–0.99).^24^ Additionally, patients with CHH with anosmia and/or pathological genetic variants had lower LH responses to kisspeptin compared to men with CHH with variants of uncertain significance or with no genetic abnormality identified.^24^ This suggests a correlation between phenotype severity and response to kisspeptin. Notably, up to one fifth of patients with CHH can undergo reversal.^25^, ^26^ To detect this, typically it involves withdrawing hormonal replacements periodically and evaluating for evidence of spontaneous re‐emergence of HPG activity. In some centers, frequent blood sampling to assess LH pulsatility is conducted to assess for reversal; however, LH pulses can be found in patients with CHH even in the absence of reversal, and clear criteria for “normal pulsatility” to indicate reversal in this setting is lacking. Of note, patients with CHH who undergo reversal, regain their ability to respond to kisspeptin (confirming re‐emergence of functional hypothalamic GnRH neurons).^27^ Importantly, the ability to respond to kisspeptin did not depend on whether sex steroid replacement was withdrawn or not,^28^ suggesting that kisspeptin administration could be a useful and efficient test for identification of CHH for diagnosis, and for assessment of current hypothalamic state during monitoring.

### 2.2 Constitutional delay of growth and puberty

Initiation of puberty follows a normal distribution and can be regarded as “delayed” if it does not commence by the expected age according to population norms, typically by 14 years in boys. The commonest cause is CDGP,^19^, ^29^ which is a self‐limiting condition where puberty is delayed but progresses spontaneously.^29^ An important differential is CHH, whereby puberty does not commence spontaneously by the age of 18 years. Differentiating CHH and CDGP at time of first presentation can be challenging because of similar clinical presentations, especially in the absence of “red flag” features associated with CHH such as anosmia or synkinesia.^18^, ^19^, ^29^ In addition, the genetic basis of CDGP and CHH have some shared genetic architecture, although they are generally distinct, with CDGP predominantly being polygenic whereas CHH is more typically monogenic or oligogenic.^29^, ^30^, ^31^ Families and the affected child may experience significant distress because of the uncertainty of whether puberty will commence spontaneously.

At present, CHH is confirmed if puberty has not commenced spontaneously by the age of 18 years, but it is possible that reliable early identification of CHH could direct differential treatment than in CDGP, such as consideration of GnRH pump therapy or use of gonadotropins to safeguard future fertility.^22^ Unfortunately, biochemical tests to date have demonstrated insufficient sensitivity and specificity to distinguish the two.^18^, ^29^ Basal gonadotropin levels, as well as GnRH stimulation tests (with measurements of stimulated gonadotropin levels) have limited specificity. Whilst patients with CHH typically have lower LH responses to GnRH than in CDGP (as their pituitary glands have not previously been exposed to endogenous GnRH and been primed), in up to 30% of CHH patients, peak LH responses may be indistinguishable from those in CDGP.^18^, ^32^ Several GnRH receptor agonists, which have increased potency and induced more prolonged stimulation have been studied; however, they still lacked specificity with overlapping responses.^18^, ^32^ Inhibin B, which is released by Sertoli cells of the testes in response to gonadotropins, and is a marker of Sertoli cell number and function, is also typically lower in CHH compared to CDGP because of lack of minipuberty in CHH, but again it does not fully discriminate the two.^19^

The ability of the response to KP as a predictive tool to determine if children with delayed puberty have either underlying CDGP or CHH was recently evaluated in a study of 16 children with delayed puberty.^29^ The children were first “primed” with pulsatile GnRH therapy to ensure pituitary sensitivity, and then the LH response to kisspeptin‐10 was found to be 100% sensitive and specific as a marker for eventual spontaneous progression through puberty during a 4‐year follow‐up.^29^ By comparison, this study found that neither basal, nor GnRH‐stimulated, gonadotropin secretion, nor inhibin B levels in boys, nor genetic testing of pathogenic variants in 30 CHH genes, could accurately predict eventual progression through puberty.^29^ This finding is consistent with the key role of the hypothalamus in initiating puberty and the ability of kisspeptin to evaluate hypothalamic function. Whilst this study's findings are promising, larger scale investigation of the ability of KP to act as a discriminator between CHH and CDGP is required.

Given that a bolus of KP54 induces a larger LH rise than an equimolar bolus of KP10,^8^ KP54 could theoretically better differentiate CHH and CDGP than KP10; however, further study is required. Overall, kisspeptin likely represents a useful probe of hypothalamic GnRH neuronal function and has utility in the identification of CHH, monitoring for reversal of CHH, and in differentiating CHH from CDGP in patients with delayed puberty.

## 3 WOMEN WITH ANOVULATORY DISORDERS

### 3.1 Polycystic ovary syndrome

PCOS is the commonest endocrine disorder in women, affecting 10%–13% of those of reproductive age.^33^ Clinical diagnosis by updated Rotterdam criteria requires two of the following three features: oligo‐anovulation, clinical or biochemical hyperandrogenism, and polycystic ovarian morphology (PCOM).^34^ Notably, diagnosis of PCOS is often delayed and most women do not receive a diagnosis within 6 months of presentation to a healthcare professional.^35^ The pathophysiology of PCOS entails not only ovarian, but neuroendocrine dysfunction; GnRH pulse frequency is approximately 40% higher in women with PCOS compared to controls.^36^, ^37^, ^38^ High‐frequency GnRH pulse secretion preferentially stimulates pituitary LH secretion while suppressing FSH secretion through upregulation of co‐repressors.^39^ Elevated LH stimulates excessive androgen synthesis in ovarian thecal cells,^40^ which in combination with relative FSH deficiency, induces follicular arrest, accumulation of antral follicles, and PCOM.^41^

Diminished sensitivity to negative feedback of E2 and progesterone on GnRH pulsatility has been identified in women with,^42^, ^43^ and animal models of,^44^ PCOS. This effect is androgen‐dependent as evidenced by flutamide‐mediated androgen receptor (AR) blockade re‐establishing normal suppression of LH by sex steroids in women with PCOS.^45^ GnRH neurons do not express the AR, progesterone receptor (PR), or estradiol receptor‐alpha (ERα).^46^ Impaired sex steroid feedback on GnRH pulsatility in PCOS is likely mediated by neuropeptidergic inputs from ARC KNDy neurons, which express these receptors.^47^, ^48^ Indeed, enhanced ARC kisspeptin immunoreactivity and *Kiss1* expression have been identified in rodents^49^ and greater KNDy soma size was observed in a PCOS model in sheep^50^ induced by prenatal androgenization. Furthermore, targeted inhibition of ARC kisspeptin‐expressing neurons in a mouse model of PCOS lowered LH pulse frequency and restored normal androgen concentrations.^51^ In addition, circulating kisspeptin concentrations are consistently higher in PCOS compared to healthy controls; however, the source of this kisspeptin is unclear and may not necessarily be hypothalamic as kisspeptin could be produced from other tissues such as adipose tissue.^52^

The response to kisspeptin in women with PCOS has yet to be described in the literature. Existing data regarding hypothalamic *Kiss1R* expression in animal models of PCOS are scarce. Gonadotropin responses to KP appear to be preserved in rodent models of PCOS; subcutaneous boluses of KP54 induced comparable LH and FSH responses in PCOS‐like and control rats.^53^ In contrast, data suggest preserved LH, but not FSH, responses to 7‐h IV infusion of KP10,^54^ and 21‐day subcutaneous administration of KP10 in women in PCOS.^53^

### 3.2 Functional hypothalamic amenorrhea

FHA is second commonest cause of anovulation characterized by an acquired suppression of the physiological pulsatile GnRH secretion, in the absence of an identifiable anatomical or organic cause.^55^ Endocrine evaluation in FHA characteristically reveals hypogonadotropic hypogonadism, typically with low LH, preserved or low FSH, and low E2 levels less than 50 pg/mL.^55^, ^56^ Suppression of the HPG axis in FHA can be attributed to stressors including reduced energy availability because of disordered eating, weight loss, excessive exercise, and psychological stress.^55^ Hypothalamic KP neurons represent a common final pathway for mechanisms mediating the suppression of GnRH pulsatility in FHA.^57^ Psychosocial, metabolic, or immune/inflammatory stressors contribute to development of FHA^58^ through activation of the hypothalamic–pituitary–adrenal (HPA) axis.^59^ Corticotropin‐releasing hormone (CRH) is secreted largely by hypothalamic hypophysiotropic neurons in the paraventricular nucleus (PVN) and acts on CRH receptors (CRHR)^60^ to increase adrenocorticotropic hormone (ACTH) secretion from the anterior pituitary, which in turn drives glucocorticoid synthesis and secretion from the adrenal zona fasciculata.^60^ Activation of the HPA axis is associated with suppression of GnRH pulsatility; women with FHA have hypercortisolemia,^61^, ^62^ and the magnitude of cortisol elevation correlates with the degree of GnRH and LH pulse frequency suppression.^59^ Intravenous infusion of CRH reduces gonadotropin levels in women without affecting the response to GnRH stimulation.^63^ However, GnRH neurons do not express CRHRs,^64^, ^65^ again consistent with an indirect upstream mediation of this effect. Indeed, CRH and glucocorticoid receptors are expressed on ARC *Kiss1* neurons, and these neurons could act as an intermediary between the HPA and HPG axes.^66^ Supporting this, exposure of female mice to psychosocial, metabolic, and immune stress reduces hypothalamic *Kiss1* expression^67^ and ARC *Kiss1* neuron activation,^68^ as does administration of CRH or corticosterone.^67^ In women with FHA, there is a negative correlation between serum cortisol and the pulse frequency of kisspeptin.^69^ Thus, hypercortisolemia secondary to various stressors in FHA leads to suppression of hypothalamic kisspeptin secretion and downstream HPG axis activation.

From an evolutionary perspective, metabolic status is thought to regulate reproductive function given the energy cost of reproduction.^55^ Acute caloric restriction in healthy women increases HPA axis activity and reduces LH pulsatility.^70^, ^71^ Chronic caloric restriction, as occurs in women with anorexia nervosa (AN), also leads to an abnormal LH secretion pattern.^72^, ^73^ LH pulse frequency decreases once energy availability falls below a threshold of 30 kcal/kg lean body mass (LBM) per day; restriction to 20 or 10 kcal/kg LBM suppressed LH pulse frequency by 16% and 39%, respectively.^74^ The threshold of energy availability at which menstrual disturbance occurs is more variable, likely reflective of differential genetic predisposition in affected individuals.^75^ Women with FHA have lower fasting circulating leptin levels, and an absence of the normal diurnal leptin variation compared to weight‐ and body‐composition‐matched controls.^76^, ^77^ Administering recombinant leptin to women with FHA increased LH pulsatility and restored ovarian activity in some women, suggesting that leptin can restore pulsatile GnRH secretion.^78^ Ghrelin is an orexigenic peptide secreted by the stomach and is also reflective of energy status.^56^ Ghrelin levels were significantly elevated in FHA compared to normally menstruating women (648.4 ± 92.0 vs. 596.7 ± 79.0 pg/mL).^79^ Furthermore, ghrelin administration to healthy women reduces gonadotropin secretion, suggesting that elevated ghrelin could be contributing to suppression of GnRH pulsatility observed in FHA.^80^

Women with FHA had significantly lower circulating kisspeptin levels than healthy controls.^81^ They also demonstrate a four‐fold greater LH response to acute administration of 6.4 nmol/kg IV KP54 compared to healthy women in the follicular phase of menstrual cycle (mean AUROC of LH secretion at 4 h following kisspeptin injection 40.2 vs. 9.8 h.iU/L, *p* < 0.01).^82^, ^83^ This may be because of increased hypothalamic sensitivity to kisspeptin secondary to *Kiss1r* upregulation, as has been demonstrated in fasting rats.^84^ This differential response opens the potential for using a KP stimulation test to aid in the identification of FHA; this is pertinent given the difficulty in differentiating FHA and PCOS, which are the commonest diagnoses in oligomenorrheic women.

## 4 FUNCTIONAL HYPOGONADAL HYPOGONADISM IN MEN

Men appear more resistant to disruption of the HPG axis from physiologic stressors than women.^85^ Studies have shown reduced serum testosterone levels in response to exercise^86^, ^87^, ^88^, ^89^ and to caloric restriction,^90^, ^91^, ^92^ although with levels remaining within the normal range, and inconsistent reports with regards to reduction in LH concentration and pulsatility.^86^, ^87^, ^88^, ^89^ As gonadotropin concentrations are not increased in response to a low testosterone, that is, secondary hypogonadism, this suggests hypothalamic/pituitary dysfunction. In male rats, fasting reduces serum testosterone concentrations and circulating LH; however, testicular responses to human chorionic gonadotropin (hCG) and pituitary responses to GnRH were unaffected, in keeping with a hypothalamic etiology.^93^ Consistent with this, pulsatile IV GnRH infusion in young men prevents the fasting‐induced decline in LH secretion, whilst also restoring serum total and free testosterone concentrations,^94^ consistent with overcoming hypothalamic deficiency.

Although the male reproductive axis is more robustly defended in the face of insufficient energy availability, case series have described functional hypogonadotropic hypogonadism (FHH) with symptomatic hypogonadism (decreased libido, reduced frequency of morning erections, and erectile dysfunction).^95^, ^96^ FHH is analogous to FHA in women, and includes people with relative energy deficiency in sport (RED‐S), a condition seen in athletes where a negative energy balance suppresses reproductive function.^97^ A more recent study looking at 10 men with FHH found no difference in LH pulse frequency compared to a control group but significantly lower LH pulse amplitude.^85^ A longitudinal study of men with FHH showed some reversibility of their HPG axis suppression upon restoring a more physiologic energy/caloric balance in a similar manner to women with FHA.^85^ Given the paucity of data regarding the neuroendocrine features of FHH in men, further studies are warranted to confirm the mechanisms involved.

Low leptin levels may be implicated in the pathophysiology of male FHH in a similar fashion to female FHA, leading to HPG axis suppression. Leptin administration attenuates the reduction in LH pulsatility and serum testosterone seen in fasting men.^98^ Ghrelin administration to healthy men suppressed LH pulse frequency and amplitude,^99^ as in female FHA. A study of low energy availability in six men, defined as low caloric intake relative to exercise‐induced energy expenditure, showed a significant reduction in leptin and insulin, but did not impact ghrelin or testosterone levels.^100^ Hypercortisolemia is evident in patients with low calorific intake and overtraining, as in FHA, and increased HPA axis activity may suppress the male HPG axis.^101^, ^102^, ^103^ The concept that thresholds of bodyweight below which gonadal axis function is impacted in humans has been largely explored in women, but with more limited evidence in men.^103^ Hypothetically, stimulated LH responses to kisspeptin in men with FHH could be increased (analogous to increased responses in FHA),^82^ but data investigating this are awaited.

## 5 LATE‐ONSET HYPOGONADISM

Circulating testosterone levels decline with increasing age in men. Historical population‐based studies identified decreases in serum testosterone of 1%–2% per year after the third decade onwards.^104^, ^105^ However, large aggregate datasets from Europe and Australia have since estimated a smaller decline of approximately 0.5% per year.^106^, ^107^ The onset of this effect may also occur later than previously believed; a recent meta‐analysis of 4075 men demonstrated minimal changes in testosterone between the ages of 17 and 70 years, but a significant annual decrease from the age of 70 years onwards.^108^ While circulating testosterone levels remain within the normal range in most older men,^107^ some develop a mild deficiency. The combination of biochemical hypoandrogenism with clinical signs or symptoms of hypogonadism, including sexual, or erectile dysfunction, decreased bone mass, muscle weakness, cognitive decline and low mood, is defined as late‐onset hypogonadism (LOH).^109^

The pathophysiology of LOH remains incompletely understood. Existing evidence implicates diminished Leydig cell mass and function in older men. Leydig cell mass in men of age 50–76 years was 44% lower than in those of age 20–48 years.^110^ Aged Leydig cells display defective LH‐stimulated steroidogenesis in vitro.^111^ Furthermore, testosterone responses to pulsatile infusion of recombinant LH following suppression of endogenous GnRH are lower in older men than in young controls.^112^, ^113^, ^114^, ^115^ Taken together, these data suggest that primary gonadal failure plays a significant role in the pathogenesis of LOH.

However, subtle compensatory but insufficient elevations in LH levels with aging have been identified in population‐based studies suggesting hypothalamo‐pituitary dysfunction.^106^, ^107^ Patterns of LH release differ between healthy, non‐obese older men and young controls; older men exhibit LH pulses of higher frequency,^115^, ^116^ lower amplitude,^115^, ^117^ and greater disorganization.^115^, ^116^ Given that GnRH administration dose‐dependently evokes comparable pituitary LH responses in older men and young controls,^118^ the LH release patterns seen in older men likely reflect abnormal hypothalamic GnRH secretion into the hypophyseal‐portal circulation. Administration of graded ganirelix (a competitive GnRH receptor antagonist) was used to indirectly assess endogenous GnRH tone and indirectly reflect hypothalamic function. A two‐fold greater suppression of LH pulses was demonstrated in older men than in young controls,^117^ implying greater availability of GnRH receptors and thus, inferentially, a relative paucity of GnRH secretion in the older cohort. An ensemble model integrating ganirelix responses with LH and testosterone pulsatility data from 18 healthy men aged 50–75 years identified deficits not only in Leydig cell responses to LH, but GnRH release and sensitivity to androgen feedback, highlighting the multiple complex reproductive changes during healthy aging.^117^ Indeed, aging men displayed aberrant LH responses to disruption of androgen feedback; ketoconazole‐induced suppression of androgenesis led to blunted increases in LH pulse amplitude in older men compared to healthy young men.^116^ Furthermore, the restorative effects of transdermal testosterone repletion on LH pulse regularity in androgen‐suppressed men diminishes with age, and was abolished by GnRH bolus administration,^119^ implying that impaired testosterone feedback occurs at the hypothalamus rather than the pituitary. Testosterone exerts its effects directly, via AR and, following local aromatization to E2, via ERα.^120^ While GnRH neurons express neither AR nor ERα,^121^ upstream ARC KNDy neurons highly co‐express these receptors,^122^ and transcriptional activity of KNDy neurons is inhibited by testosterone.^122^ Interestingly, there are decreases in AR expression, but not ERα expression, in the ARC of aged rhesus macaques.^123^ Thus, age‐related changes in KNDy neuron receptor expression and stimulation by androgens could result in the dysregulated GnRH pulse generation observed in older men, contributing to age‐related hypogonadism.

Kisspeptin offers the opportunity to interrogate hypothalamic function in older men. Comparable LH responses to bolus administration of KP10 (1 µg/kg) and low‐dose (0.1 and 0.3 nmol/kg/h) KP54 infusion have been observed in older men and young controls.^13^, ^124^ In contrast, higher dose KP54 infusion (1.0 nmol/kg/h) elicited greater LH secretion in men over 50 years old.^13^ While disparities in isoform, half‐life, duration of post‐intervention observation, and sampling frequency preclude direct comparison of these studies, the differential LH responses observed at KP54 doses of 1.0 nmol/kg/h likely represent near‐maximal stimulation of GnRH neurons. Exaggerated responses to this dose in older men could reflect age‐related decline in endogenous hypothalamic KP and corresponding upregulation of *Kiss1R* on hypothalamic GnRH neurons. Thus, altered LH responsivity to KP in older men corroborates existing data indicating mixed gonadal and hypothalamic deficits in aging men. Future studies investigating responses to KP in older men, both with and without obesity, may shed further light on the hypothalamic component of LOH.

## 6 OBESITY‐RELATED SECONDARY HYPOGONADISM

Obesity is an important risk factor for hypogonadism in both men and women.^125^, ^126^ Similarly to age‐related hypogonadism, obesity likely has both a primary effect on the testes by negatively impacting Leydig cell function, as well as a secondary effect on the HPG axis.^127^ Male obesity‐related secondary hypogonadism (MOSH) is relatively well characterized,^15^ and evidence suggests that a similar non‐PCOS female obesity‐related secondary hypogonadism (FOSH) also exists.^14^ Visceral adiposity, leptin resistance, and insulin resistance are proposed pathophysiological factors in obesity‐related HPG axis dysfunction.^128^

A study of eight men with obesity showed a reduction in LH pulse amplitude, but no change in LH frequency compared to normal weight controls.^129^ Increased adiposity is associated with an increase in E2 levels in MOSH, owing to greater adipose tissue aromatase activity, which is proposed to suppress GnRH and LH concentrations through negative feedback at the hypothalamus and pituitary.^130^, ^131^ Correspondingly, treating MOSH men with the aromatase inhibitor letrozole normalizes serum testosterone, which could in part reflect reduced aromatization of testosterone to estrogen leaving more substrate.^130^ However, this hypothesis has been challenged by other data that showed lower circulating E2 levels in men with MOSH.^132^

In FOSH, androgen concentrations are increased,^133^ with a positive association between free testosterone levels and visceral adipose tissue.^134^ There is increased conversion of androstenedione to testosterone via 17β‐hydroxysteroid dehydrogenase 5 (also known as aldo‐keto reductase family 1 member C3, AKR1C3) in subcutaneous fat, with expression of the enzyme positively correlating with BMI (*r* = 0.506), albeit this enzyme has a preference for the alternative rather than classic pathway of androgen synthesis.^135^ In female rats, testosterone administration reduced serum LH levels and suppressed hypothalamic KP mRNA expression.^136^ In humans, 6‐h IV administration of testosterone to supraphysiological levels in lean healthy women reduced both LH pulse frequency and amplitude above a certain threshold (421 ng/dL); this effect appeared to be independent of aromatization to estrogen.^137^ A further study showed that increasing testosterone levels by three‐fold over a 12‐h period in healthy women (to 120 ng/dL) resulted in approximately 50% increase in LH pulsatile secretion, but when testosterone levels were raised by six‐fold (to 245 ng/dL), mean LH fell by 22% because of a fall in basal LH.^138^ Thus, small increases in androgens may be stimulatory to GnRH pulsatility in women without PCOS, but higher levels above a threshold could contribute to a reduction in the LH levels.^182^ Leptin indirectly stimulates hypothalamic GnRH secretion through upstream hypothalamic KP neurons to regulate the reproductive axis in normal conditions.^139^ Individuals with obesity demonstrate hyperleptinemia but also leptin resistance,^140^ which results in hypogonadism through reduction of kisspeptin neurons and in turn GnRH neurons.^141^ Several mechanisms are thought to contribute to leptin resistance in states of hyperleptinemia: saturated leptin transport through the blood–brain barrier,^142^ activation of feedback circuits in the presence of chronic overstimulation of LepR inhibiting downstream signaling pathways,^143^ impaired leptin receptor trafficking, saturation of leptin signaling pathways, and endoplasmic reticulum stress.^144^ Male mouse models of diet‐induced obesity demonstrate reduced plasma LH and testosterone levels alongside reduced hypothalamic expression of *GnRH, Kiss1, Kiss1R*, and *LepR*.^145^ This suggests that leptin resistance mediates hypothalamic dysregulation of the reproductive axis through suppression of kisspeptin signaling.

Insulin resistance causing hyperinsulinemia is observed in obesity.^125^ Mice with neuron‐specific deletion of insulin receptors have increased body mass and hypogonadotropic hypogonadism, confirming the importance of insulin signaling in the brain for normal reproductive axis activity.^146^ Insulin resistance is thought to impair hypothalamic KP neuronal function and downstream reproductive axis activity.^15^ Streptozotocin‐induced diabetic rats with hypoinsulinemia have reduced GnRH secretion^147^, ^148^ and reduced ARC *Kiss1* mRNA expression.^149^ However, GnRH‐specific and KP‐specific insulin receptor knockout models both have preserved oestrus cyclicity and fertility,^150^, ^151^, ^152^ suggesting that insulin does not directly modulate reproductive function through these neurons. Low circulating testosterone levels are associated with insulin resistance in MOSH.^125^ In females, hyperinsulinemia resulting from insulin resistance is linked to increased androgen levels^153^; insulin stimulates both ovarian^154^ and adipocyte androgen production,^155^ whilst decreasing hepatic synthesis of sex hormone‐binding globulin (SHBG),^156^ leading to increased circulating free androgens.

Pro‐inflammatory cytokines released by adipocytes also suppress KP signaling. Rabbits fed a high‐fat diet showed increased hypothalamic IL‐6 expression and reduced *Kiss1R* expression.^157^ Further, in vitro evidence from human fetal hypothalamic cell lines showed that TNF‐α reduced GnRH secretion, *Kiss1R* expression, and kisspeptin‐induced depolarizing effect.^158^

Thus, suppression of hypothalamic KP signaling appears to have a fundamental role in the pathophysiology of obesity‐related secondary hypogonadism. This has been demonstrated by administering IV KP10 to obese hypogonadal men, resulting in increased serum LH and testosterone levels as well as increased LH pulsatility, thereby overriding the hypothalamic deficit in KP signaling.^159^ In male rats with streptozocin‐induced diabetes, the LH response to kisspeptin was significantly higher than control animals,^160^ suggesting possible increased sensitivity. It would be interesting to evaluate if this augmented LH response to kisspeptin translated to obesity‐related secondary hypogonadism in men with obesity. Further studies examining the response to kisspeptin in patients with obesity could help determine the contribution of hypothalamic dysfunction in obesity‐related hypogonadism.

## 7 HYPERPROLACTINEMIA

Prolactin is a pleiotropic peptide hormone secreted by the lactotroph cells of the anterior pituitary gland,^161^ with effects on lactogenesis, the stress response, energy expenditure, and reproduction.^162^ Prolactin autoregulates its release through a feedback loop with tubero‐infundibular dopaminergic (TIDA) neurons of the hypothalamus; binding of prolactin to its receptor on TIDA neurons stimulates dopamine release into the hypophyseal‐portal circulation, activation of dopamine‐2 receptors (D2R) on the lactotroph cell membrane, and suppression of intracellular prolactin synthesis.^161^, ^163^ Hyperprolactinemia may be physiological or pathological. Physiological hyperprolactinemia arises from adaptive processes such as lactation^163^; in contrast, pathological hyperprolactinemia occurs secondary to autonomous prolactin secretion or disruption of auto‐feedback.^163^, ^164^ Prolactin‐secreting pituitary adenomas (prolactinomas) represent the commonest cause of pathological hyperprolactinemia, accounting for 40% of all presentations.^165^ Other causes include: “disconnection” hyperprolactinemia, in which pituitary stalk compression by a non‐functioning pituitary adenoma or hypothalamic craniopharyngioma interrupts dopaminergic inhibition of prolactin synthesis^166^; dopaminergic neuronal damage following infiltration or irradiation of the hypothalamus; systemic diseases affecting dopaminergic tone; and pharmacological modulation of dopamine activity.^167^ Clinically, hyperprolactinemia is characterized by a hypogonadotropic hypogonadism associated with erectile dysfunction and reduced libido in men, oligo‐amenorrhea in women, and infertility and loss of bone mass in both sexes.^164^

Prolactin exerts suppressive effects on pulsatile LH secretion. Exogenous prolactin dose‐dependently reduces LH pulse frequency and amplitude in gonadectomized male^168^ and female^169^, ^170^ rats. Similarly, observational studies of hyperprolactinemic men and women have reported absent or low‐frequency LH pulse secretion, which was reversed by restoration of normal prolactin levels.^171^, ^172^, ^173^ Comparable LH responses to exogenous GnRH observed in hyperprolactinemic women and controls indicate preserved sensitivity and secretory function of pituitary gonadotrophs.^173^, ^174^ In contrast, rats bearing prolactin‐secreting pituitary allografts displayed decreased hypophyseal‐portal GnRH concentrations^175^ and a diminished post‐castration rise in hypothalamic GnRH mRNA expression^176^ compared to normoprolactinemic controls. Furthermore, subcutaneous GnRH replacement can re‐establish follicular development in women with hyperprolactinemic amenorrhea.^171^ Under 5% of GnRH neurons express the prolactin receptor^177^; therefore, prolactin likely inhibits hypothalamic GnRH release through an intermediate mechanism.

Hypothalamic KP neurons of the ARC and rostral periventricular area of the third ventricle (RPV3) have emerged as putative intermediaries for prolactin's suppressive effects on GnRH release. Both neuronal populations abundantly express the prolactin receptor,^177^, ^178^ and exhibit “responsivity” to exogenous prolactin.^178^, ^179^ Reduced *Kiss1* mRNA expression and kisspeptin immunoreactivity have been demonstrated in the ARC, and to a lesser extent the RPV3, of ovariectomized rodents receiving exogenous prolactin^170^, ^180^ and lactating dams^181^ compared to normoprolactinemic controls. Consistent with an underlying deficit in hypothalamic kisspeptin secretion, daily intraperitoneal injections of KP10 restored normal serum LH levels and ovarian cyclicity in prolactin‐treated mice.^180^ Similarly, both continuous and pulsatile infusion of KP10 increased mean serum LH and pulse frequency in hyperprolactinemic women.^182^, ^183^ Abolition of prolactin's suppressive effects on LH pulsatility following conditional knockout of its receptor in the mouse ARC^184^ suggests that ARC KP neurons represent the principal target for prolactin's effects on the HPG axis. Interestingly, E2 may modulate prolactin's effects on ARC‐KP neurons; E2 itself downregulates *Kiss1* expression in the ARC^48^ and potentiates prolactin's effects on LH pulse frequency and amplitude in ovariectomized rats.^170^ Collectively, these data suggest that prolactin exerts partially E2‐dependent effects on ARC‐KP neurons to suppress kisspeptin, and ultimately downstream GnRH release.

Gonadotropin responses to kisspeptin may offer further insight into the functional status of GnRH and KNDy neurons in hyperprolactinemia. Prolactin‐mediated suppression of ARC KP release may result in upregulation of hypothalamic *KISS1R* expression; hypothetically, this should result in augmented gonadotropin responses to KP. To date, no studies in humans have quantified gonadotropin responses to KP in both hyper‐ and normoprolactinemic patients, although studies have shown that women with hyperprolactinemia still respond to a kisspeptin‐10 infusion.^185^ In rats, 10‐fold higher integrated LH responses to kisspeptin infusion have been observed during lactation than in dioestrus.^186^ Furthermore, greater hypothalamic *KISS1R* expression has been demonstrated in adult male rats with PTU‐induced hypothyroidism and consequent hyperprolactinemia than in controls.^187^ However, intrinsic damage sustained by hypothalamic GnRH neurons or pituitary gonadotrophs because of common etiologies of hyperprolactinemia, such as invasive pituitary and hypothalamic tumors,^188^ may attenuate or abrogate gonadotropin responses to kisspeptin in these conditions. Thus, combined dynamic testing with kisspeptin and GnRH may illuminate the functional status of hypothalamic KNDy and GnRH neurons, as well as pituitary gonadotrophs, in hyperprolactinemia.^188^

## 8 STRUCTURAL BRAIN PATHOLOGY

Structural brain pathology such as tumors, or treatments such as surgery or irradiation, may cause damage to the hypothalamus, pituitary, or both, leading to secondary hypogonadism. Whilst some conditions disproportionately affect hypothalamic function, such as hamartomas and germinomas,^189^ others can affect both the hypothalamus and pituitary, such as craniopharyngiomas (CPs). CPs are rare tumors with a bimodal age distribution at presentation, with peaks in childhood and in older age.^190^ CPs often affect the HPG axis: LH and FSH deficiencies are seen in 40%–50% of cases at diagnosis,^191^ and secondary hypogonadism leads to absent puberty in up to 70% of children with CP.^192^ Both surgical resection and radiotherapy for CPs cause significant morbidity because of damage to the hypothalamus and/or pituitary gland, with long‐term consequences including hypopituitarism, hypothalamic obesity, vision loss, and motor deficits.^193^ Radiotherapy is particularly a risk factor for central hypogonadism at doses above 50 Gy.^192^

CPs were previously described as “suprasellar” because of the presence of calcification visible above the sella turcica on skull radiographs.^190^ The resulting compression on the pituitary was thus presumed to be the cause of central hypogonadism, and the hypothalamus was assumed to be spared. However, more recently, MRI has demonstrated that approximately half of CPs are in fact located within the regions containing the hypothalamus, at the level of the third ventricle, and thus termed “hypothalamic CPs (Hy‐CPs).”^194^ Although hypogonadotropic hypogonadism secondary to CP has traditionally been presumed to be pituitary in origin,^195^ following the discovery of Hy‐CPs, Pascual et al. postulated that they may cause three different syndromes of clinical manifestations: (i) pan‐hypopituitarism because of blockage of hypothalamic releasing hormones including GnRH at the level of the portal vessels; (ii) infundibulo‐tuberal syndrome caused by damage to the median eminence; and (iii) hypothalamic syndrome (HS) from damage to hypothalamic structures along the third ventricle wall.^190^ Thus, hypogonadotropic hypogonadism seen in CP may be because of pituitary and/or hypothalamic damage; however, these two groups cannot be differentiated based on basal or stimulated pituitary hormone profiles. Imaging can be useful to help determine the extent of hypothalamic involvement of a CP and the amount of damage sustained during resection,^196^, ^197^ and van Santen et al. developed a diagnostic tool incorporating clinical signs and symptoms, endocrine dysfunction, and MRI findings to determine the presence of HS in children, including those with tumors such as CPs.^198^ However, imaging is unable to determine hypothalamic and pituitary endocrine function, nor differentiate these two causes of hypogonadism.

Kisspeptin could be used to interrogate hypothalamic function in patients with structural brain pathology and hypogonadotropic hypogonadism. In parallel, the response to GnRH would elicit any associated pituitary involvement. However, to date, no studies have been carried out to this end. This may be useful to monitor any change in hypothalamic reproductive function over time, given that the incidence of central hypogonadism increases for years following cranial irradiation for brain malignancies, whilst conversely there could be recovery of hypothalamic function after an insult.^199^, ^200^

## 9 CONCLUSION

In conclusion, kisspeptin offers a potential novel diagnostic avenue for elucidating the relative hypothalamic contribution to many different reproductive disorders in males and females. In age‐ and obesity‐related hypogonadism, the hypothalamic (secondary) component is postulated to lead to increased sensitivity to kisspeptin and thus greater gonadotropin response, allowing differentiation from the testicular (primary) component. Similarly, comparing gonadotropin responses to kisspeptin and gonadotropin‐releasing hormone stimulation could help distinguish hypothalamic from pituitary involvement in structural brain pathology, as well as determine the specific abnormality in the different causes of hyperprolactinemia. Kisspeptin could also enable clinicians to distinguish congenital hypogonadotropic hypogonadism from constitutional delay of growth and puberty in children with delayed puberty. Finally, in functional hypogonadotropic hypogonadism, including functional hypothalamic amenorrhea in women and relative energy deficiency syndrome in both sexes, kisspeptin could identify reduced hypothalamic function as the final common pathway in the pathophysiology of these conditions. It could also potentially help differentiate these conditions from differential diagnoses such as polycystic ovary syndrome, as a prompt and accurate diagnosis has implications for clinical management and improved patient care.

To date, there are multiple avenues that remain under‐explored regarding the potential use of kisspeptin in interrogating hypothalamic dysfunction in both pubertal and reproductive disorders. For example, data on the response to kisspeptin in men with functional hypothalamic hypogonadism such as relative energy deficiency syndrome, or in patients with structural brain pathology would be of interest. Additionally, work investigating whether the response to kisspeptin could reveal differential underlying hypothalamic dysfunction in women with distinct causes of menstrual disturbance would also be of value given the significant diagnostic challenge in current clinical practice. A limitation of the use of kisspeptin is that it relies on a functional pituitary gland to enable a readout with respect to gonadotropin secretion, as gonadotropin‐releasing hormone levels cannot be measured directly. In future, kisspeptin could be used in practice as a routine test of hypothalamic function, just as we currently regard gonadotropin‐releasing hormone as a standard test of pituitary function. The ability to interrogate hypothalamic dysfunction is likely to reveal novel insights in several reproductive disorders given its central role in integrating metabolic and reproductive health.

## AUTHOR CONTRIBUTIONS

**Aureliane C. S. Pierret**, **Aaran H. Patel** and **Elisabeth Daniels**: Performed the literature review and wrote the paper. **Ali Abbara**, **Waljit S. Dhillo** and **Alexander N. Comninos**: Edited the manuscript and provided supervision.

## CONFLICT OF INTEREST STATEMENT

Ali Abbara and Waljit S. Dhillo have consulted for Myovant Sciences Ltd. Waljit S. Dhillo has consulted for KaNDy therapeutics.

## DISCLOSURE

The views expressed are those of the authors and not necessarily represent those of the MRC, the NHS, the NIHR, or the Department of Health.

## Data Availability

The data that support the findings of this study are available from the corresponding author upon reasonable request.
